# Serial Interval and Incubation Period Estimates of Monkeypox Virus Infection in 12 Jurisdictions, United States, May–August 2022

**DOI:** 10.3201/eid2904.221622

**Published:** 2023-04

**Authors:** Zachary J. Madewell, Kelly Charniga, Nina B. Masters, Jason Asher, Lily Fahrenwald, William Still, Judy Chen, Naama Kipperman, David Bui, Meghan Shea, Katharine Saunders, Lori Saathoff-Huber, Shannon Johnson, Khalil Harbi, Abby L. Berns, Taidy Perez, Emily Gateley, Ian H. Spicknall, Yoshinori Nakazawa, Thomas L. Gift, 2022 Mpox Outbreak Response Team

**Affiliations:** Centers for Disease Control and Prevention, Atlanta, Georgia, USA (Z.J. Madewell, K. Charniga, N.B. Masters, J. Asher, I.H. Spicknall, Y. Nakazawa, T.L. Gift);; Chicago Department of Public Health, Chicago, Illinois, USA (L. Fahrenwald);; District of Columbia Department of Health, Washington, DC, USA (W. Still);; New York City Department of Health and Mental Hygiene, New York, New York, USA (J. Chen, N. Kipperman);; California Department of Public Health, Sacramento, California, USA (D. Bui);; Colorado Department of Public Health and Environment, Denver, Colorado, USA (M. Shea);; Florida Department of Health, Tallahassee, Florida, USA (K. Saunders);; Illinois Department of Public Health, Springfield, Illinois, USA (L. Saathoff-Huber);; Michigan Department of Health and Human Services, Lansing, Michigan, USA (S. Johnson);; North Carolina Department of Health and Human Services, Raleigh, North Carolina, USA (K. Harbi);; Rhode Island Department of Health, Providence, Rhode Island, USA (A.L. Berns);; South Carolina Department of Health and Environmental Control, Columbia, South Carolina, USA (T. Perez);; Tennessee Department of Health, Nashville, Tennessee (E. Gateley)

**Keywords:** monkeypox, mpox, viruses, sexually transmitted infections, outbreak response, parameter estimation, epidemiology, natural history, United States, monkeypox virus

## Abstract

Using data from 12 US health departments, we estimated mean serial interval for monkeypox virus infection to be 8.5 (95% credible interval 7.3–9.9) days for symptom onset, based on 57 case pairs. Mean estimated incubation period was 5.6 (95% credible interval 4.3–7.8) days for symptom onset, based on 35 case pairs.

Since May 6, 2022, mpox (formerly monkeypox) cases have been reported across the globe. According to the Centers for Disease Control and Prevention (CDC), 85,115 confirmed mpox cases and 182 deaths have occurred in 110 locations across historically endemic and nonendemic regions as of January 25, 2023 (*1*). Mpox symptoms usually start within 3 weeks of exposure to monkeypox virus (MPXV) and may include fever, headache, chills, swollen lymph nodes, and exhaustion (*2*). A rash usually develops within 1–4 days after onset of symptoms. MPXV is transmitted through close contact with infectious rash, scabs, or body fluids; respiratory droplets during prolonged face-to-face contact; and fomites such as clothing, towels, or bedding (*1*). Transmission in the current outbreak has occurred primarily through close physical contact associated with sexual activities among gay, bisexual, and other men who have sex with men. Transmission of MPXV is possible from the time of symptom onset until all scabs have fallen off and fully healed (*3*).

The serial interval is defined as the time between symptom onset in a primary case-patient and symptom onset in the secondary case-patient and depends on the incubation period (the time from a person’s infection to the onset of signs and symptoms) (*4*), epidemic phase, and population contact patterns. The serial interval is critical for estimating the effective reproduction number (R_t_) and forecasting incidence, both of which are important for understanding the course of an outbreak and the effect of interventions (e.g., antiviral drugs and vaccines). In the current outbreak, many patients report multiple anonymous sex partners or attendance at large events, such as festivals, in the 3 weeks before symptom onset, which has complicated efforts to identify primary and secondary case pairs. By using preliminary data from 17 mpox case pairs in the United Kingdom, researchers estimated the mean serial interval to be 9.8 days with high uncertainty (95% credible interval [CrI] 5.9–21.4 days) (*5*). An investigation of 16 primary and secondary case pairs in Italy indicated the estimated mean generation time, or time between infection of primary and secondary cases, to be 12.5 (95% CrI 7.5–17.3) days (*6*). In this report, we estimate the serial interval and incubation period for symptom onset and rash onset for MPXV infection in the United States.

## The Study

Data on self-reported symptom and rash onset dates for primary and secondary case pairs, including the type of contact that occurred between pairs, were compiled by 12 state and local health departments. We examined serial interval for both earliest symptom onset and rash onset because the latter may be more specific to mpox than the other signs. Earliest symptom onset included any mpox symptom as defined by CDC (*2*), including rash. We only included cases if there was a high degree of certainty that the secondary case-patient was infected by the primary case-patient (Appendix).

For each case pair, we calculated days between onset of any mpox symptoms and days between rash onset in the primary and secondary case-patients. We used the EpiEstim package version 4.1.2 in R software (The R Foundation for Statistical Computing, https://www.r-project.org) to estimate the distribution of the serial interval for known primary and secondary case pairs using Bayesian methods for symptom and rash onset (Appendix) (*7*). We did not adjust for right-truncation of the data because we included cases during the stable or declining phase of the outbreak (*8*).

**Figure 1:**
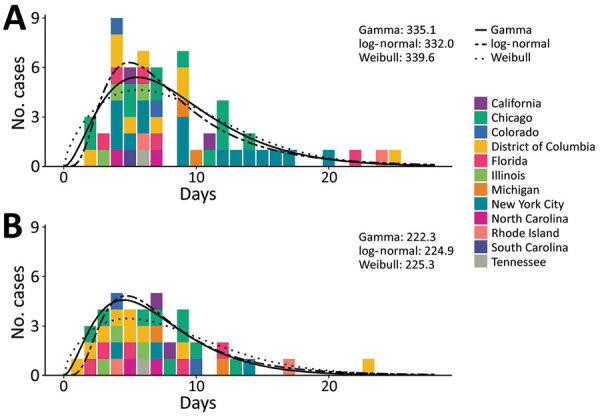
Empirical and fitted distributions of the serial intervals of rash onset (A) (n = 40 cases) and symptom onset (B) (n = 57 cases) for monkeypox virus, 12 jurisdictions, United States, May–August 2022. Leave-one-out information criterion values for each model are shown inside the plots in the upper right-hand corner.

We received data for 120 case pairs from 13 jurisdictions (Appendix Figures 1, 2). Fifty-seven case pairs met the inclusion criteria (Figure 1; Appendix). Dates of symptom onset among primary case-patients ranged from May 11 to August 13, 2022 (Appendix Figure 3). Forty of the 57 pairs included rash onset dates for primary and secondary case-patients. We also considered in our analysis the type of contact for the case pairs (Appendix Table 1). The gamma distribution provided the best fit to the serial interval data. The overall mean estimated serial interval for symptom onset was 8.5 (95% CrI 7.3–9.9) days (SD 5.0 [95% CrI 4.0–6.4] days) and for rash onset was 7.0 (95% CrI 5.8–8.4) days (SD 4.2 [95% CrI 3.2–5.6] days) (Table; Appendix Tables 2, 3).

**Table:** Estimated incubation period and serial interval of monkeypox virus infection, 12 jurisdictions, United States, May–August 2022*

Onset	Incubation period, d				Serial interval, d		
	Mean (95% CrI)	SD (95% CrI)	No. cases		Mean (95% CrI)	SD (95% CrI)	No. case pairs
Symptom onset	5.6 (4.3–7.8)	4.4 (2.8–8.7)	36		8.5 (7.3–9.9)	5.0 (4.0–6.4)	57
Rash onset	7.5 (6.0–9.8)	4.9 (3.2–8.8)	35		7.0 (5.8–8.4)	4.2 (3.2–5.6)	40

*CrI, credible interval.

**Figure 2:**
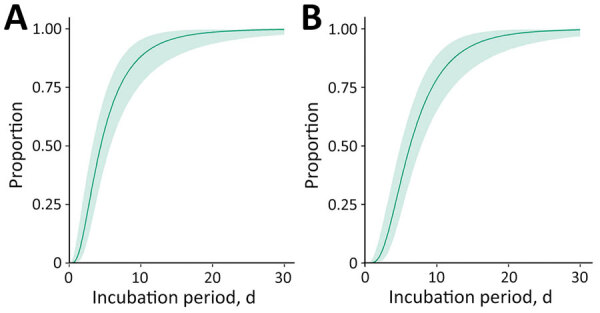
Updated estimated cumulative density functions according to a log-normal distribution of monkeypox virus incubation periods, by symptom onset (A) (n = 36 cases) and rash onset (B) (n = 35 cases), 12 jurisdictions, United States, May–August 2022.

We estimated the incubation period using 22 US mpox cases reported in an earlier study (K. Charniga et al., unpub. data, https://doi.org/10.1101/2022.06.22.22276713) plus 14 cases from our dataset (Appendix). Of the new case-patients, 10 were exposed during a single day. The mean incubation period from exposure to symptom onset for 36 case-patients was 5.6 (95% CrI 4.3–7.8) days (SD 4.4 [95% CrI 2.8–8.7] days), whereas the mean incubation period from exposure to rash onset for 35 case-patients was 7.5 (95% CrI 6.0–9.8) days (SD 4.9 [95% CrI 3.2–8.8] days) (Figure 2; Appendix Figure 4).

## Conclusions

Determining the serial interval of a pathogen can inform our understanding of the timing of transmission relative to symptom onset. The serial interval of 8.5 days for symptom onset was similar to an estimate from the United Kingdom of 9.8 days after correcting for right-truncation (*5*) but shorter than the generation time of 12.5 days reported from Italy (*6*). The serial interval correlates with human behavior and can decrease with increasing awareness among men who have sex with men or interventions, a pattern similar to that observed among the general population during the COVID-19 pandemic (*9*). The Italy estimates were from the initial period of the epidemic (May–June 2022), whereas our study was for July–August 2022. Serial interval changes can be very rapid (*10*). We also found a serial interval of 7.0 days for rash onset. The estimated serial interval for symptom onset was longer than that for the incubation period (5.6 days), suggesting most transmission occurred after the onset of symptoms in the primary case-patient. Conversely, the serial interval for rash onset (7.0 days) was slightly shorter than that for the rash incubation period (7.5 days), which may suggest some prerash transmission; indeed, there were instances in the observed data where secondary case-patients were exposed before onset of reported rash in the primary case-patient. However, the credible intervals for the estimates overlap. The serial interval for symptom onset ranged from 2 to 25 days. This wide range may be attributable in part to variability in the nature and intensity of contact.

The first limitation of this study is that precise ascertainment of symptom and rash onset dates is critical for serial interval estimation, but initial mpox symptoms are often nonspecific and may be unrelated to MPXV infection. Second, despite careful selection of linked primary and secondary case pairs, exposure from additional unknown sources may have occurred. Third, social desirability bias may have factored into the self-reported exposures before infection. Fourth, serial interval may vary by age, underlying conditions, vaccination status, or contact type (route of exposure); we did not stratify our analysis by these factors because of limited data. Fifth, we excluded secondary case-patients who had symptom onset on the same day as or before the primary case-patient to ensure a high degree of confidence linking case pairs; however, the serial interval could be negative (*11*). Sixth, the serial interval for rash onset could be biased if rash is more quickly identified in the secondary case-patient because of case finding and investigation of the primary case-patient. 

Notwithstanding those limitations, our estimate of the serial interval for MPXV infection includes more case pairs than have been reported previously from the United Kingdom (*5*) and Italy (*6*). We also provide estimates for rash onset, which may be more reliable than initial symptom onset for determining serial interval.

Appendix Additional information about serial interval and incubation period estimates of monkeypox virus infection in 12 jurisdictions, United States, May–August 2022.
