# Supplementary material for: Serial Interval and Incubation Period Estimates of Monkeypox Virus Infection in 12 Jurisdictions, United States, May–August 2022
Source: Emerg Infect Dis. 2023 Apr;29(4):818–21. doi: 10.3201/eid2904.221622 (PMC10045696; doi:10.3201/eid2904.221622)
Supplement: Appendix — Additional information about serial interval and incubation period estimates of monkeypox virus infection in 12 jurisdictions, United States, May–August 2022. [file 22-1622-Techapp-s1.pdf]

Article DOI: https://doi.org/10.3201/eid2904.221622

Serial Interval and Incubation Period Estimates of Monkeypox Virus Infection in 12 Jurisdictions, United States, May–August 2022

Appendix

Supplementary Methods

Serial interval data

We solicited data on known primary/secondary case pairs from public health departments across the United States. Jurisdictions were asked to provide data on first symptom onset and rash onset date for linked primary and secondary cases, as well as contact type (e.g., sexual or intimate, indirect contact). Jurisdictions were also asked to separate the serial interval data into two parts: 1) case pairs in which the exposure of the secondary case met the strict criteria below, and 2) case pairs for which there was a high degree of epidemiologic evidence that the primary case transmitted the virus to the secondary case via accepted modes of transmission but did not meet our strict criteria.

Inclusion criteria for serial interval case pairs

We defined strict criteria for case pairs to ensure the secondary case was most likely infected by the primary case. Patient interviews conducted during contact tracing confirmed that secondary cases did not: 1) report exposure to multiple potential primary cases; 2) have more than one sexual partner in the 3 weeks before symptom onset; 3) have symptom onset on the same day as the primary case; or 4) attend a festival, bath house, sex party, or other crowded event in the 3 weeks before symptom onset and had minimal or no clothing during the event.

Selection of case pairs for serial interval analysis

A small subset of all probable and confirmed mpox cases reported in the United States were provided for the serial interval analysis. Jurisdictions provided the following rationale for how case pairs were selected:

- California: the case pairs provided were some of the first cases investigated that had clear epidemiologic linkages and high suspicion for transmission (i.e., clearly reported contact between primary and secondary and secondary case patient did not have other reported exposures beyond index case)
- Chicago, Illinois, Rhode Island, and Tennessee: all cases that were positively identified as having contact to a known case were selected for review
- New York City: pairs were included if they had a high degree of certainty that the pair constituted a true primary/secondary relationship. This assessment was based on a combination of data pieces (onset date, last exposure date, exposure type) and investigation notes. Pairs were excluded if the secondary case reported multiple sexual contacts during their incubation period, had ambiguous source of exposure, or had an implausible incubation period (time from last exposure to primary to secondary case symptom onset >22 days)
- Washington DC: all positive cases were contacted for interview and ≈60% of all positive cases were able to be interviewed during the study period. Among all cases interviewed, records of each interview were retrospectively reviewed to identify close contacts. When a close contact was confirmed to be another positive case, we examined the notes and conferred with the investigator who conducted the interview to ensure that the pair constitutes a true case pair as per the study definition.

CDC recommends close contacts of mpox cases monitor themselves for signs and symptoms for a period of 21 days (*1*). We expect jurisdictions would have used this guidance to inform the duration of follow-up for contact tracing. However, secondary cases were not excluded if they were exposed more than 21 days before symptom onset.

Inclusion criteria for incubation period cases

For incubation period estimation, we added 14 US cases to the monkeypox virus (MPXV) incubation period analysis first reported in a preprint on June 23, 2022 (K. Charniga et al., unpub. data, https://doi.org/10.1101/2022.06.22.22276713). Two jurisdictions provided exposure date for the secondary case, even though it was not part of the data request for this study. In contrast to the preliminary analysis, we only included US cases (N = 22 for earliest symptom onset and N = 21 for rash onset), because we hypothesize that there may be differences across countries which could be related to the selection of cases and therefore, introduce selection bias.

Demographics of cases

Demographic data were not part of the data request for this project. We matched mpox cases in the serial interval dataset with those in the surveillance data submitted by jurisdictions to DCIPHER by case ids. DCIPHER is a cloud-based platform used by CDC and other federal, state, local, tribal, and territorial jurisdictions to collect and share public health data (*2*).

Demographic data for the 22 US cases included in the incubation period analysis were reported in Charniga et al (K. Charniga et al., unpub. data, https://doi.org/10.1101/2022.06.22.22276713).

Evidence for pre-symptomatic mpox transmission

We would expect secondary cases with longer incubation periods would also have longer serial intervals. We calculated Pearson's correlation coefficient for serial interval and incubation period in the secondary case for 15 individuals who were included in both analyses. We also evaluated the evidence for pre-symptomatic mpox transmission which is indicated when the incubation period is longer than the serial interval.

Fitting serial interval distributions

EpiEstim (*3*,*4*) requires a dataframe with five columns: the lower (EL) and upper (ER) bounds of symptom onset in the primary case, lower (SL) and upper (SR) bounds of symptom onset in the secondary case, and censoring type. EL was assumed to be at $t = 0$ and SL was the number of days between symptom onset in the primary and secondary case. ER and SR were calculated by adding 1 to the lower bounds' values. The censoring type was doubly interval-censored.

We used the R package coarsedatatools (*5*) for Bayesian parametric estimation of the serial interval distribution which uses Markov chain Monte Carlo (MCMC) methods. The likelihood of a doubly interval-censored observation is:

$$L(\theta; X_i, \lambda) = \prod_{i=1}^{n} \int_{E_{Li}}^{E_{Ri}} \int_{S_{Li}}^{S_{Ri}} h_\lambda(e) f_\theta(s-e) \mathrm{d}s\mathrm{d}e$$

Where i represents a unique case pair, n is the total number of case pairs, (E_{Ri},E_{Li}) and (S_{Ri},S_{Li}) are the intervals for symptom onset of the primary and secondary case, respectively, $h_\lambda(.)$ is the uniform probability density function (pdf) of the exposure time E, and $f_\theta(.)$ is the pdf of the serial interval, which follows one of three distributions currently supported with the function dic.fit.mcmc: log-normal, gamma, and Weibull.

This procedure produces a posterior distribution for the parameters of the selected distribution (e.g., shape and scale for gamma), with each set of parameters corresponding to a single step in the MCMC chain. We used the init_mcmc_params function which identifies starting points for MCMC, which are identified based on the observed mean and standard deviation of the difference in symptom onset data. We calculated mean and standard deviation for each set of shape and scale parameters, and subsequently calculated overall means and 95% credible intervals (CrI).

Log-normal, Weibull, and gamma distributions were fitted to the difference-in-days data. We used the loo package (*6*) in R (version 2.5.0) to calculate the widely applicable information criterion (WAIC) and leave-one-out information criterion (LOOIC). WAIC and LOOIC were used to compare fits, and the model with the lowest WAIC/LOOIC value was selected. The serial interval was estimated for symptom onset and rash onset using 50,000 Markov Chain Monte Carlo (MCMC) samples and a burn-in of 10,000 samples. Convergence of MCMC samples was assessed by the Gelman–Rubin statistic.

Fitting incubation period distributions

To estimate the incubation period, we used the same methods as those reported by Charniga et al (K. Charniga et al., unpub. data, https://doi.org/10.1101/2022.06.22.22276713). These methods were adapted from Lessler et al. (*7*) and Reich et al. (*5*). Briefly, we obtained the window of exposure to MPXV as well as time of earliest symptom onset and rash onset. We used

the exact timing of self-reported exposure. If those data were unavailable, we used information such as length of stay in a country reporting cases to bound the window of exposure. For cases with continuous household exposures, we assumed the start of the exposure for the secondary case coincided with symptom onset in the primary case. Following Lessler et al. (*7*), we bounded the time of MPXV infection by the earliest and latest potential windows of exposure.

We constructed a doubly censored dataset for the incubation period and fitted the distribution using previously described methods (*5*,*7*). We assumed the incubation period of MPXV followed a log-normal distribution and used MCMC for calibration.

Sensitivity analyses

We performed three sensitivity analyses on the serial interval distributions. The first involved including case pairs that were linked by contact tracing but did not meet our strict criteria. For the second sensitivity analysis, we did not exclude secondary cases that had symptom onset on the same day as the primary case. Finally, we used the dynamicaltruncation R package (version 0.0.0.9000) (*8*) to estimate the serial interval accounting for right truncation and interval censoring.

We also compared the definitions of clinical mpox symptoms across other studies that estimated epidemiologic distributions for the current outbreak.

Supplementary results

Serial interval data

We received 120 total case pairs with earliest symptom onset date for both primary and secondary cases from 13 jurisdictions, of which 100 pairs also had rash onset dates for both primary and secondary cases. One pair had a serial interval for rash onset of negative 1 day. We assumed this case pair had incorrectly identified the direction of transmission and reversed the order of the primary and secondary case.

Of the 120 total pairs, 63 did not meet our strict criteria: 34 had multiple sexual partners, had attended a large/crowded event, and/or were linked to more than one primary case; one pair had symptom onset on the same day; and 28 did not meet the criteria for a variety of other reasons (e.g., secondary case was not interviewed).

Demographics of cases

Out of 57 case pairs that met our strict criteria, there were 112 unique individuals (two cases were each the primary case for two secondary cases). Mean age was 35 (range 1–76). Five cases had female sex assigned at birth compared to 106 cases for which male was assigned at birth (1 case had missing information). Four cases identified as female, 105 identified as male, 1 identified as a transgender male, and 2 selected another gender identity. The age, sex, and gender identity of included cases closely matched those of all mpox cases reported in the United States as of early October 2022 (*9*).

Evidence for pre-symptomatic mpox transmission

We found a statistically significant correlation between the serial interval and incubation period in secondary cases that were included in both analyses (N = 15, Appendix Figure 4). Out of 15 secondary cases, 4 had continuous exposures to primary cases, and 1 case had an exposure window that started before symptom onset in the primary case and ended after symptom onset in the primary case. For these cases, we were not able to assess pre-symptomatic transmission on an individual level. Based on the remaining 10 case pairs, we found transmission may have occurred 1–3 days before rash onset in 50% of cases and 1 day before earliest symptom onset in 10% of cases (these cases are shown in Appendix Figure 4 as those that have error bars completely above the diagonal black line). Compared to a recent study by Ward et al. (*10*), we found attenuated evidence for pre-symptomatic mpox transmission among US case pairs.

Sensitivity analyses

For all 120 pairs (including one pair with same-day symptom onset), mean serial interval for symptom onset was 7.9 days (95% CrI 7.0–8.8) days and standard deviation (SD) was 5.1 days (95% CrI 4.3–6.1). For 100 pairs (including three pairs with same-day rash onset), mean serial interval for rash onset was 6.8 days (95% CrI 5.9–7.8) and SD was 5.0 days (95% CrI 4.1–6.1).

Excluding one case pair with same-day symptom onset, mean serial interval was 7.9 days (95% CrI 7.1–8.9) days and SD was 5.0 days (95% CrI 4.3–5.9) (119 pairs). Excluding case pairs with same-day rash onset, mean serial interval was 7.0 days (95% CrI 6.1–8.0) and SD was 4.7 days (95% CrI 3.9–5.7) (97 pairs).

Restricting to 58 case pairs that met our strict criteria (including one pair with same-day symptom onset), mean serial interval for symptom onset was 8.4 days (95% CrI 7.1–9.8 days) and SD was 5.1 days (95% CrI 4.2–6.8). Restricting to 41 case pairs that met our strict criteria (including one pair with same-day rash onset), mean serial interval for rash onset was 6.9 days (95% CrI 5.7–8.3 days) and SD was 4.3 days (95% CrI 3.3–5.8).

We found that compared to a model that adjusted for interval censoring alone, adjusting for both right truncation and interval censoring did not change the serial interval's mean for earliest symptom onset or rash onset; however, there was a small impact on the uncertainty of the SD.

We found the lists of mpox symptoms used in Miura et al., Ward et al., and Guzetta et al. were remarkably similar to the one used by CDC. Miura et al. (*11*) did not define mpox symptoms in their paper, but they cited the CDC webpage on Signs and Symptoms of mpox (*12*) in their introduction. The only notable difference between the list of mpox symptoms used by CDC and those used by Ward et al. (*10*) (citing the UK National Health Service (*13*)) and Guzzetta et al. (*14*) (citing the World Health Organization (*15*)) is that the CDC includes respiratory symptoms (e.g., sore throat, nasal congestion, or cough). If respiratory symptoms are due to another cause (such as seasonal allergies or COVID-19), we would expect a shorter incubation period among mpox cases whose first symptom was respiratory in nature. In contrast, the impact on the serial interval is less clear and could bias the estimate in either direction.

References

1. Centers for Disease Control and Prevention. What to do if you are a close contact of a person with monkeypox. 2022 [cited 2022 Nov 29]. https://www.cdc.gov/poxvirus/monkeypox/prevention/close-contact.html

2. US Department of Health and Human Services. Privacy impact assessment. 2020 [cited 2022 Nov 17]. https://www.hhs.gov/sites/default/files/cdc-dcipher-whr.pdf

3. Cori A, Cauchemez S, Ferguson NM, Fraser C, Dahlqwist E, Demarsh PA, et al. EpiEstim: estimate time varying reproduction numbers from epidemic curves. 2020 [cited 2022 Nov 23]. https://cran.r-project.org/web/packages/EpiEstim/index.html

4. Cori A, Ferguson NM, Fraser C, Cauchemez S. A new framework and software to estimate time-varying reproduction numbers during epidemics. Am J Epidemiol. 2013;178:1505–12. PubMed https://doi.org/10.1093/aje/kwt133

5. Reich NG, Lessler J, Cummings DA, Brookmeyer R. Estimating incubation period distributions with coarse data. Stat Med. 2009;28:2769–84. PubMed https://doi.org/10.1002/sim.3659

6. Vehtari A, Gelman A, Gabry J, Yao Y. loo: efficient leave-one-out cross-validation and WAIC for Bayesian models. 2021 [cited 2022 Nov 30]. https://cran.r-project.org/web/packages/loo/index.html

7. Lessler J, Ott CT, Carcelen AC, Konikoff JM, Williamson J, Bi Q, et al. Times to key events in Zika virus infection and implications for blood donation: a systematic review. Bull World Health Organ. 2016;94:841–9. PubMed https://doi.org/10.2471/BLT.16.174540

8. Abbott S, Park Sang W. Adjusting for common biases in infectious disease data when estimating distributions. 2022 [cited 2022 Nov 28]. https://github.com/parksw3/dynamicaltruncation

9. Kava CM, Rohraff DM, Wallace B, Mendoza-Alonzo JL, Currie DW, Munsey AE, et al. Epidemiologic features of the monkeypox outbreak and the public health response—United States, May 17–October 6, 2022. MMWR Morb Mortal Wkly Rep. 2022;71:1449–56. PubMed https://doi.org/10.15585/mmwr.mm7145a4

10. Ward T, Christie R, Paton RS, Cumming F, Overton CE. Transmission dynamics of monkeypox in the United Kingdom: contact tracing study. BMJ. 2022;379:e073153. PubMed https://doi.org/10.1136/bmj-2022-073153

11. Miura F, van Ewijk CE, Backer JA, Xiridou M, Franz E, Op de Coul E, et al. Estimated incubation period for monkeypox cases confirmed in the Netherlands, May 2022. Euro Surveill. 2022;27:2200448. PubMed https://doi.org/10.2807/1560-7917.ES.2022.27.24.2200448

12. Centers for Disease Control and Prevention. Monkeypox: signs and symptoms. 2022 [cited 2022 Nov 22]. https://www.cdc.gov/poxvirus/monkeypox/symptoms/index.html

13. United Kingdom National Health Service. Monkeypox. 2022 [cited 2022 Nov 28]. https://www.nhs.uk/conditions/monkeypox

14. Guzzetta G, Mammone A, Ferraro F, Caraglia A, Rapiti A, Marziano V, et al. Early estimates of monkeypox incubation period, generation time, and reproduction number, Italy, May–June 2022. Emerg Infect Dis. 2022;28:2078–81. PubMed https://doi.org/10.3201/eid2810.221126

15. World Health Organization. Multi-country monkeypox outbreak in non-endemic countries. 2022 [cited 2022 Nov 28]. https://www.who.int/emergencies/disease-outbreak-news/item/2022-DON385

Appendix Table 1. Type of contact among case pairs included in the serial interval analysis*

Type of contact	Symptom onset, N (%)	Rash onset, N (%)
Caregiving	1 (1.8)	0 (0)
Face-to-face contact, not including intimate contact	1 (1.8)	0 (0)
Healthcare	1 (1.8)	1 (2.5)
Household	2 (3.5)	2 (5.0)
Shared bedding	3 (5.3)	1 (2.5)
Sexual or intimate contact	49 (86.0)	36 (90.0)
Total	57 (100)	40 (100)

*Case pairs with more invasive exposures (i.e., sexual or intimate contact) likely had also additional types of contact.

Appendix Table 2. Distributions of estimated incubation period and serial interval of monkeypox virus, United States, May–August 2022*

	Incubation period		Serial interval	
Onset	Log mean (95% CrI)	Log standard deviation (95% CrI)	Shape (95% CrI)	Scale (95% CrI)
Symptom onset	1.5 (1.2–1.8)	0.7 (0.5–1.0)	2.9 (2.0–4.1)	2.9 (2.0–4.4)
Rash onset	1.8 (1.6–2.1)	0.6 (0.4–0.8)	2.8 (1.8–4.2)	2.5 (1.6–4.0)

*The distributions for the incubation period were log-normal, whereas the distributions for the serial interval were gamma. CrI credible interval

Appendix Table 3. Serial interval estimates and 95% credible intervals (CrI) for earliest symptom onset and rash onset using gamma, log-normal, and Weibull distributions*

Onset	Distribution	Mean (95% CrI)	SD (95% CrI)	WAIC	LOOIC
Symptom onset	Gamma	8.5 (7.3–9.9)	5.0 (4.0–6.4)	335.1	335.1
	Log-normal	7.2 (6.1–8.4)	1.8 (1.7–2.1)	332.0	332.0
	Weibull	8.6 (7.3–10.0)	5.2 (4.3–6.5)	339.5	339.6
Rash onset	Gamma	7.0 (5.8–8.4)	4.2 (3.2–5.6)	222.2	222.3
	Log-normal	6.7 (5.5–8.0)	1.8 (1.6–2.1)	224.8	224.9
	Weibull	8.0 (6.5–9.7)	5.1 (4.1–6.7)	225.3	225.3

*While gamma was the best fit for rash onset, log-normal was slightly preferred for symptom onset. Because the differences in WAIC/LOOIC were small, we reported gamma for both in the main text. WAIC: widely applicable information criterion; LOOIC: leave-one-out information criterion

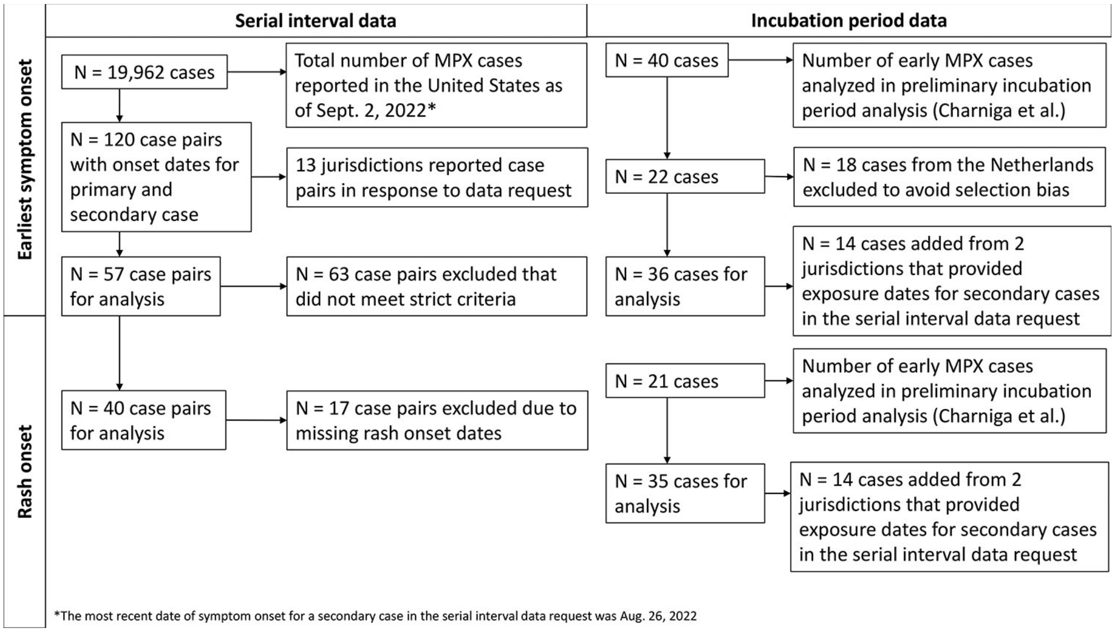

Appendix Figure 1. Selection criteria for cases included in both the serial interval and incubation period analysis.

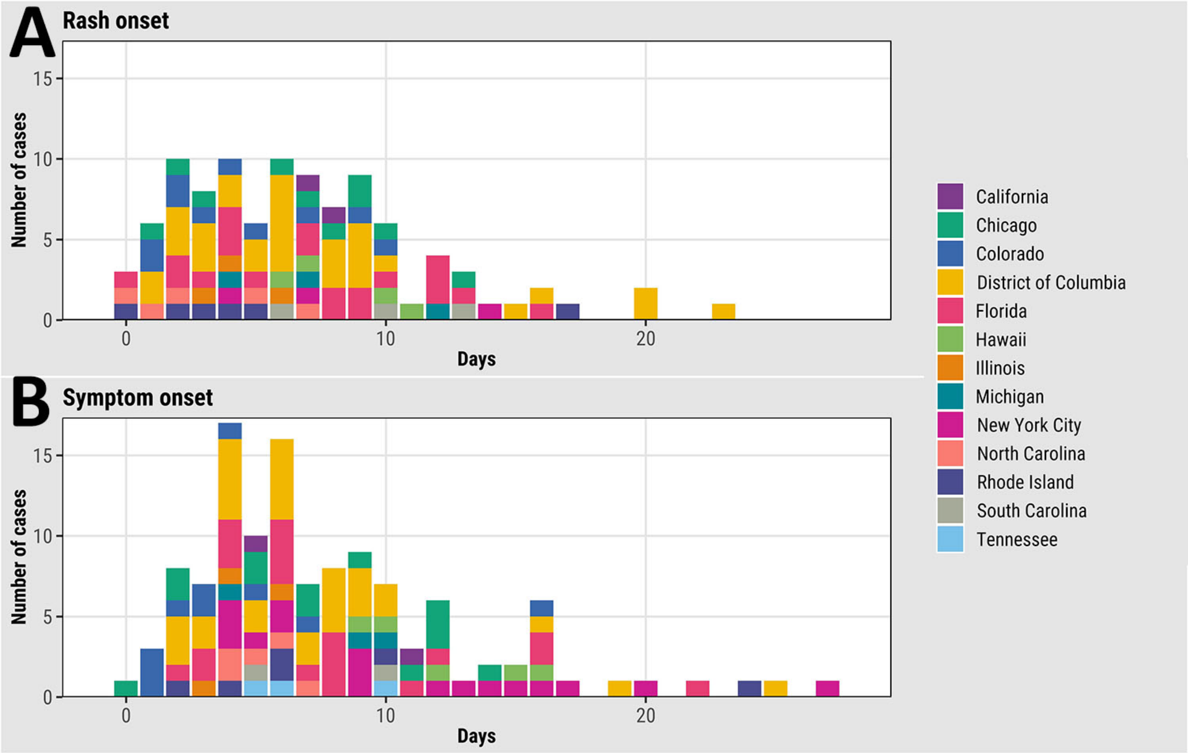

Appendix Figure 2. Empirical distribution of the serial intervals for rash (N = 100) and symptom (N = 120) onset for monkeypox virus for all case pairs, United States, May–August 2022. Colors represent US jurisdictions (N = 13).

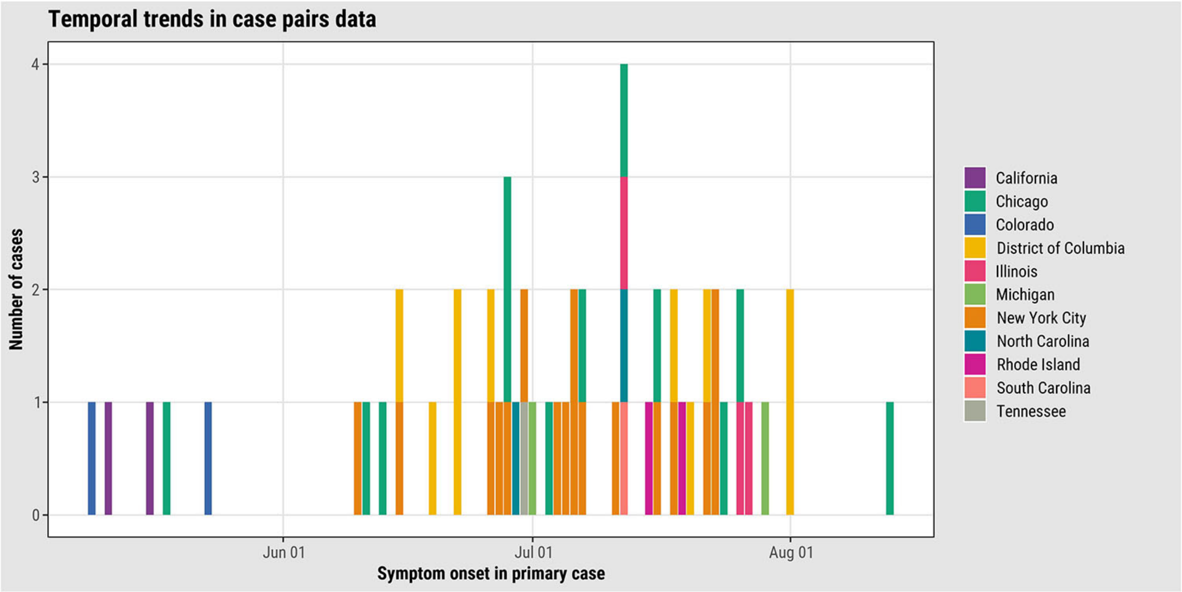

Appendix Figure 3. Temporal trends in monkeypox case pairs data, United States, May–August 2022.

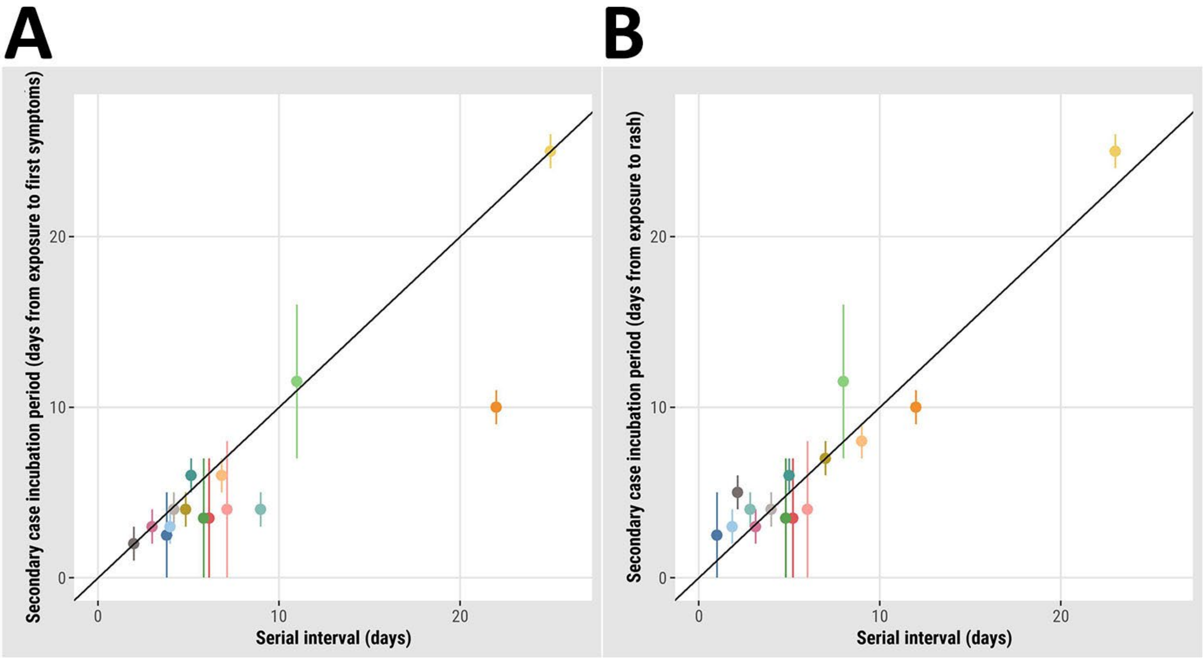

Appendix Figure 4. Correlation between the serial interval (SI) and incubation period in secondary cases that were included in both analyses (N = 15), United States, May–August 2022 and evidence for pre-symptomatic MPXV transmission. The error bars represent the possible range of the incubation period for each secondary case for (A) earliest symptom onset and (B) rash onset. The points are colored according to case id. Pearson's correlation coefficient was calculated for the midpoint between the minimum and maximum incubation period for each secondary case. The correlation was 0.88 ($p < 0.001$) for earliest symptom onset and 0.93 ($p < 0.001$) for rash onset. The black reference line is $y = x$.
